# The Application of Ribosome Engineering to Natural Product Discovery and Yield Improvement in *Streptomyces*

**DOI:** 10.3390/antibiotics8030133

**Published:** 2019-08-30

**Authors:** Saibin Zhu, Yanwen Duan, Yong Huang

**Affiliations:** 1 Xiangya International Academy of Translational Medicine at Central South University, Changsha 410013, Hunan, China; 2 Hunan Engineering Research Center of Combinatorial Biosynthesis and Natural Product Drug Discovery, Changsha 410011, Hunan, China; 3 National Engineering Research Center of Combinatorial Biosynthesis for Drug Discovery, Changsha 410011, Hunan, China

**Keywords:** ribosome engineering, strain improvement, natural products, *Streptomyces*

## Abstract

Microbial natural product drug discovery and development has entered a new era, driven by microbial genomics and synthetic biology. Genome sequencing has revealed the vast potential to produce valuable secondary metabolites in bacteria and fungi. However, many of the biosynthetic gene clusters are silent under standard fermentation conditions. By rational screening for mutations in bacterial ribosomal proteins or RNA polymerases, ribosome engineering is a versatile approach to obtain mutants with improved titers for microbial product formation or new natural products through activating silent biosynthetic gene clusters. In this review, we discuss the mechanism of ribosome engineering and its application to natural product discovery and yield improvement in *Streptomyces*. Our analysis suggests that ribosome engineering is a rapid and cost-effective approach and could be adapted to speed up the discovery and development of natural product drug leads in the post-genomic era.

## 1. Introduction 

Streptomycetes are proficient producers of bioactive natural products, such as antibiotics (streptomycin and daptomycin), anticancer agents (bleomycin and doxorubicin), immunosuppressants (rapamycin), and agents used in veterinary medicine and agriculture (avermectins and spinosyn) [1,2,3]. Many *Streptomyces* natural products or their derivatives are also essential tools to discover new biology [4]. Rapamycin, a macrocyclic polyketide discovered from *Streptomyces hygroscopicus* in 1972, simultaneously binds FKBP12 and the mammalian target of rapamycin (mTOR) [5]. These studies led to the discovery of mTOR complex 1 and mTOR complex 2, which regulate protein synthesis or cellular metabolism in mammalian cells [6]. Several rapalogs, derivatives of the parent rapamycin, are useful in the treatment of renal cell carcinoma [7]. Rapamycin-inspired macrocycles recently became drug leads for the treatment of kidney ischaemia reperfusion injury [8]. 

**Figure 1:**
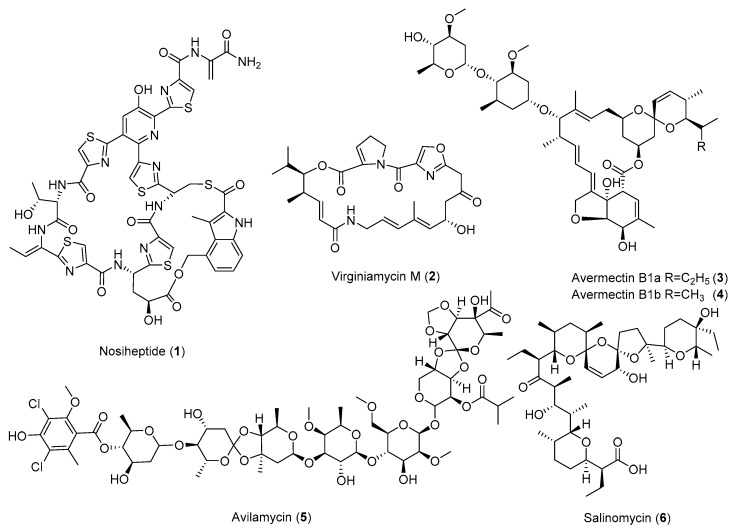
Representative structures of overproduced compounds useful in agriculture and veterinary medicine through ribosome engineering.

**Figure 2:**
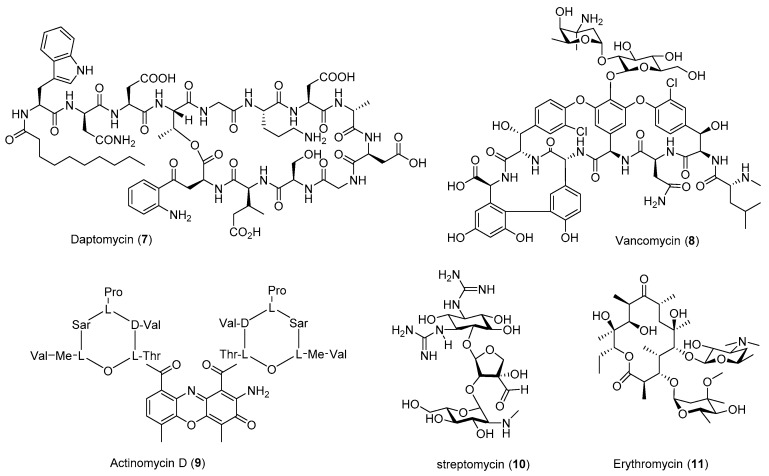
Representative structures of overproduced clinical medicines through ribosome engineering.

The genome sequences of *Streptomyces avermitilis* and *Streptomyces coelicolor* A3(2) reveal that they contain 25 or 22 biosynthetic gene clusters (BGCs), respectively [9,10]. These surprising findings at the beginning of the 21st century suggest that most BGCs are silent under standard culture conditions and that there is a huge biosynthetic potential in the genus of *Streptomyces*. Since there are over 11,500 actinobacterial genomes listed in the Joint Genome Institute genome databases and the Genbank, the current discovered natural products from *Streptomyces* or *Actinomyces* are only the tip of an iceberg [11,12,13]. In addition, there is a huge need to increase the yield of many approved natural products-based drugs, and many of them are on the list of essential medicines recommended by the World Health Organization, such as the antibiotics vancomycin, streptomycin, and ivermectin, the semisynthetic derivative of avermectins, as well as antitumor agents bleomycin, dactinomycin, and daunorubicin [14] (Figure 1 and Figure 2). The shortage of these medicines could precipitate a major public health crisis in any modern society. 

Ribosome engineering is an approach to discover microbes with certain spontaneous mutations in their ribosome or RNA polymerase, through screening antibiotic-resistant mutants on Petri dishes. Some selected mutants may have elevated secondary metabolite production or produce new series of natural products with interesting biological activities. Ochi and co-workers discovered a streptomycin-resistant TK24 strain in the study of the model *Streptomyces* strain *Streptomyces lividans* in 1996 [15]. This natural mutant has a K88N mutation in the *rpsL* gene, which encodes ribosomal protein S12 and produces the well-studied blue pigment antibiotic actinorhodin (Act) (**23**). They and others were subsequently able to use a streptomycin screen to discover other mutations in *rpsL* in different *Streptomyces* strains. Rifampicin was later used to screen for mutants with elevated secondary metabolite production and spontaneous mutation in the *rpoB* gene encoding the RNA polymerase β subunit. Apart from mutations conferring resistance to streptomycin or rifampicin, other antibiotics, such as gentamicin, paromomycin, geneticin, fusidic acid, thiostrepton, and lincomycin, have also been successfully applied to screen for mutants with elevated levels of secondary metabolite production.

In the past two decades, this approach has been widely used for increasing the production of bioactive molecules in many bacterial species and for activating silent or poorly expressed BGCs in the post-genomic era. Recently, there have been several excellent reviews to discuss various aspects of ribosome engineering and synthetic biology approaches to engineer cellular metabolism [16,17,18]. In this short review, we focus on the discussion of the applications and mechanism of ribosome engineering to natural product discovery and yield improvement in *Streptomyces*. Our analysis suggests that ribosome engineering is a rapid and cost-effective approach and could be adapted to speed up the discovery and development of natural product drug leads in the post-genomic era. 

## 2. Application of Ribosome Engineering to Increase Antibiotic Production

**Figure 3:**
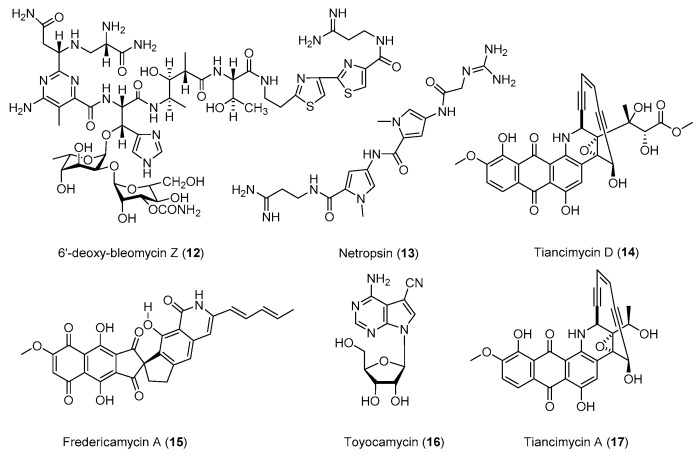
Representative structures of natural products with promising activities against tumor cells.

**Figure 4:**
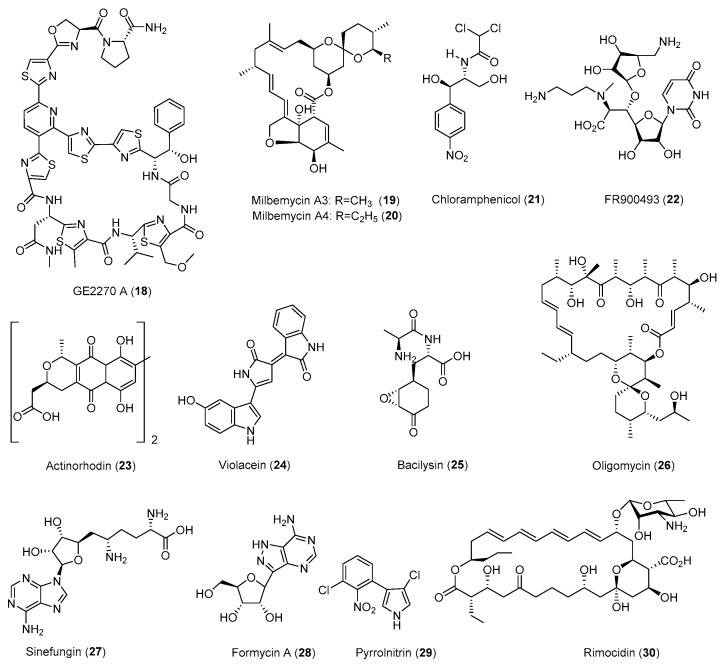
Representative natural products with antibacterial activities.

Many *Streptomyces* strains isolated from nature have low yields of the produced natural products under laboratory fermentation conditions. Thus, these strains typically need significant optimization for pilot- or industrial-scale production. Traditional strain improvements may involve mutagenesis using chemical mutagens or physical methods such as UV or γ-irradiation [19]. Many of the current strain improvement methods include genome shuffling [20,21], heterologous expression [22], and metabolic pathway engineering [23]. The emerging tools in synthetic biology would completely refactor the whole BGC of certain natural products, using the designed genetic parts and circuits [17,18]. This could be done even on a genomic scale using de novo synthetic DNAs [24]. All these emerging technologies would contribute to our understanding of the whole metabolic cellular network of given cells, which would enable the creation of a cell factory for the precise control of gene expression and the biosynthesis of a given product. However, most of these methods would require the understanding and establishment of a genetic system in the targeted organism and sophisticated molecular biology techniques. The application of ribosome engineering is rather straightforward, involving the screening and isolation of the mutants resistant to certain antibiotics (Table 1 and Table 2 and Figure 1, Figure 2, Figure 3 and Figure 4). 

### 2.1. Single Drug Resistance Mutation

Act from *S. coelicolor* or its close relatives has been one of the model compounds for the study of the enzymology of bacterial type II polyketide synthases. Ochi et al. have used several antibiotics, such as streptomycin, paromycin, and erythromycin, to improve Act production (Table 1). For example, in a streptomycin-resistant (*str*) mutant with an *rpsL* R86P mutation in *S. coelicolor* 1147 (KO-944), the production of Act reached 133.8 mg/L, at least a 55-fold increase over the parent strain [30]. Actinomycin D (**9**) (also named dactinomycin), isolated from *Streptomyces parvullus*, is a clinically used chromopeptide antineoplastic agent against trophoblastic neoplasms, testicular cancer, and several other cancers. In *S. parvullus* and *Streptomyces antibioticus*, several *str*, gentamycin-resistant (*gen*), or rifampicin-resistant (*rif*) mutants were obtained, which resulted in the multiple-fold increase of the yield of this important compound [32,33,34]. 

Enediynes are the most cytotoxic natural products known to date, and they are excellent payloads for antibody–drug conjugates against various cancers. Tiancimycins were recently discovered as 10-membered anthraquinone-type enediynes (Figure 3) [47]. The yield of tiancimycins in the wild-type *Streptomyces* sp. CB03234 was below 1 mg/L, which significantly hampered preclinical studies using this strain. A *rif* strain CB03234-R-16 and a *str* strain CB03234-S were obtained by treating the *S.* sp. CB03234 wild-type strain with rifampicin or streptomycin [48,49]. The CB03234-R-16 has an *rpoB* L422P mutation, while CB03234-S has an *rpsL* K43N mutation. The yield of tiancimycin A (**17**) in CB03234-R-16 was 22.5 ± 3.1 mg/L in shaking flasks and 13 ± 1 mg/L in 15-L fermentors. The yield of tiancimycin A (**17**) in CB03234-S reached 13.7 ± 0.3 mg/L in 25-L fermentors. Interestingly CB03234-S produced tiancimycin D (**14**), an analog of tiancimycin A (**17**), with a decent yield of 19.2 ± 0.4 mg/L. The increase in the production of these promising anticancer drug leads would provide ample compounds for the future preclinical study. 

### 2.2. Combinations of Drug Resistance Mutation

In *S. coelicolor* A3 (2), the *str/gen* or *str/rif* double mutants have a 1.7 to 2.5-fold higher ability to synthesize Act than the individual *str*, *gen*, or *rif* mutants [27,28]. Likewise, the *str/gen/rif* triple mutant and an octuple mutant C8 produced Act more than 48- or 180-fold higher than the wild-type strain, respectively [29]. Toyocamycin (**16**) is an important member of the nucleoside antibiotic family with diverse biological activities. As a promising antibiotic, it may have broad utility to control plant diseases. Yu and co-workers systematically increased the yield of toyocamycin (**16**) in *Streptomyces diastatochromogenes* 1628 by screening for *rif* or *str/str/par* mutants, which afforded strains 1628-T15 with an *rpoB* H437R mutationand SD3145 with a truncation mutation in RsmG [51]. The yield of toyocamycin (**16**) increased to 0.68 and 1.5 g/L in strains 16228-T15 and SD3145, respectively. Salinomycin (**6**), a polyether antibiotic produced by *Streptomyces albus*, is used as a coccidiostat in chicken feed. It was recently shown to reduce epithelial cancer stem cells >100-fold over paclitaxel and is thus a usefuldrug lead against cancer stem cells [59]. The titer of salinomycin (**6**) was increased to 25 g/L in a *str/gen/rif* triple mutant strain of *S. albus* KO606, a remarkable 2.3-fold increase relative to the starting industrial strain. This strain contains an *rpsL* K88N mutation, while no mutation was identified in *ropB* [45]. Similarly, the titer of a polyene macrolide rimocidin in *S. rimosus* increased over 4-fold compared with the wild-type strain, with >0.6 g/L in a 5-L fermentor [44].

### 2.3. Combination of Traditional Mutagenesis and Ribosome Engineering 

Ribosome engineering was also frequently used in combination with traditional physical and chemical breeding methods to obtain mutants of higher yields. Sinefungin (27) is a nucleoside antibiotic with strong antifungal, antiviral, and anti-trypanosome activity. In *Streptomyces incarnatus* NRRL 8089, optimized UV-irradiation and *rif* screening resulted in a mutant strain, rif-400, with its production increased by 7-fold [46]. This strain has a single mutation A1340G in the *rpoB* gene, which corresponds to a D447G mutation. Bleomycins, glycopeptide antitumor antibiotics, are clinically used to treat various malignancies in combination chemotherapy. 6′-Deoxy-bleomycin Z (12) is a novel bleomycin derivative discovered through combinatorial engineering of the BGCs of bleomycin and zorbamycin from *Streptomyces verticillus* ATCC 15003 and *Streptomyces flavoviridis* SB9001, respectively. By combined UV mutagenesis, antibiotic screening with *gen*, *str*, or *rif*,as well as fermentation optimization, a mutant strain *S. flavoviridis* G4F12 was obtained. It produced **12** with a titer above 70 mg/L under the optimized fermentation conditions, representing a 7-fold increase over its original production [53]. 

### 2.4. Combination of Genome Shuffling and Ribosome Engineering

Genome shuffling is an approach involving recursive genomic recombination within a population of phenotypically selected bacteria to generate new strain libraries, which may result in pronounced improvement in the screened phenotype. The combination of genome shuffling and ribosome engineering has been widely used in antibiotic development, including avilamycin (**5**) [36], daptomycin (**7**) [39], nosiheptide (**1**) [43], and virginiamycin (**2**) [52]. For example, avilamycin (**5**), a feed industry antimicrobial agent approved by the European Union, is used to inhibit the growth of multidrug resistant Gram-positive bacteria. Lv et al. [36] combined genome shuffling with *str* screening to obtain an improved recombinant strain *Streptomyces viridochromogenes* E-219. The yield of avilamycin in this strain reached 1.4 g/L, a 36.8-fold increase in comparison with that of the ancestor *S. viridochromogenes* strain. 

### 2.5. Overexpression of Ribosome Recycling Factor 

At the end of protein translation in living cells, ribosome recycling factor encoded by the *frr* gene participates in ribosome recycling. Overexpression of *frr* in several *Streptomyces* strains led to increased production of Act, avermectins (**3** and **4**), and toyocamycin (**16**). By overexpressing the ribosome recycling factor in the avermectin producer, the yield of avermectin increased by 3–3.7 times to over 8 g/L [35]. Similarly, the overexpression of a ribosome recycling factor also increased the yield of toyocamycin (**16**) to about 0.6 g/L due to the increased protein synthesis in the late-growth phase of the *Streptomyces* mycelium [50]. 

### 2.6. The Application of Ribosome Engineering in Other Bacteria and Fungi 

Ribosome engineering has also been used in other bacterial species, including several *Bacillus* strains, *Escherichia coli*, and *Paenibacillus agaridevorans*, and in some fungi. For example, the introduction of three drug-resistance mutations in *rsmG*, *rpsL*, and *rpoB* in *P. agaridevorans* markedly enhanced the productivity of cycloisomaltooligosaccharide glucanotransferase (CITase) by more than 1100-fold as compared to the wild-type strain [60]. Ahmetagic et al. [58] introduced lincomycin- and kanamycin-resistance into *E. coli* K12 AA23 pPSX-vioABCDE opv-1 to produce 41-fold higher expression of violacein (**24**). Finally, ribosome engineering is used not only to increase the production of secondary metabolites but also to increase the yield of α-amylase [61], xylanase [62], vitamins [63], ε-poly-L-lysine [64,65,66,67], L-isoleucine [68], and fuels including butanol [69] and ethanol [70] (Table 3). 

## 3. Discovery of New Natural Products Using Ribosome Engineering 

Genome sequencing has revealed the vast potential to produce valuable secondary metabolites in *Streptomyces*. However, many BGCs are silent under standard fermentation conditions. Natural product synthesis in *Streptomyces* is affected by many factors, including nutrient supply including carbon and nitrogen sources, cofactors, the expression of functional biosynthetic enzymes, drug resistance, and export mechanisms. Many approaches have been used in activating silent BGCs [71,72], such as the “one strain many compounds” approach [73,74], cocultivation [75,76], manipulation of pleiotropic or pathway-specific regulators, genome mining [77,78], and BGC refactoring [17]. 

**Figure 5:**
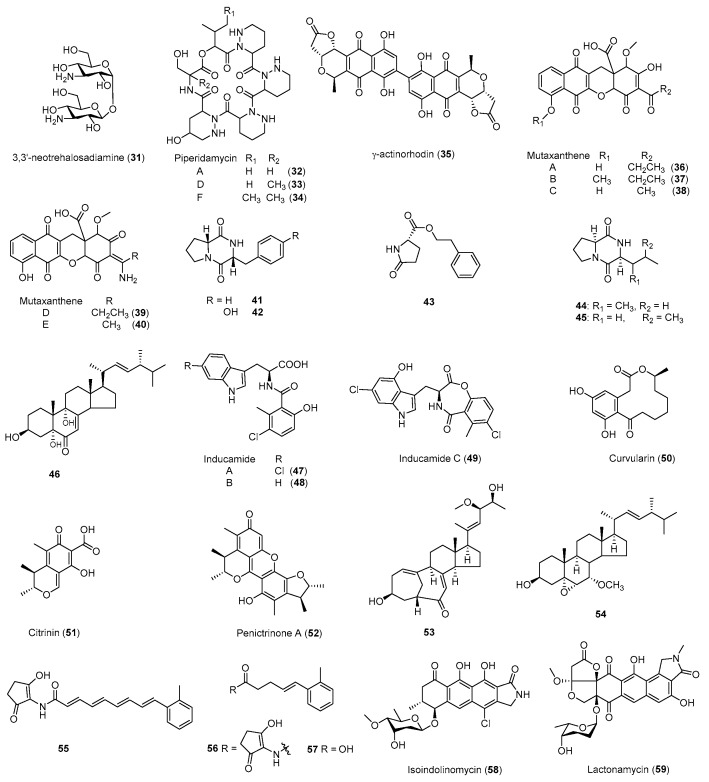
Representative natural products discovered by ribosome engineering.

In 2009, Ochi and coworkers performed massive antibiotic-resistance screening on 1068 actinomycetes from soil, using rifampicin or streptomycin [79]. They discovered that 43% (51/119) of the nonantibiotic-producing *Streptomyces* strains from soil were able to commence antibacterial production. This could be due to the marked enhancement of previously undetectable antibiotic production or to triggering the biosynthesis of new antibiotics (Table 4). They subsequently isolated a series of novel macrocyclic piperidamycins (**32**–**34**) (Figure 5) from one soil strain *Streptomyces mauvecolor* with either a single H437L mutation in *rpoB* or double mutation in *rpoB* (H437L) and *rspL* (K88R). They proposed that the enhanced gene expression for piperidamycin biosynthesis in the mutants was due to the increased affinities of mutant RNA polymerases for their promoters, based on surface plasmon resonance analysis and in vitro synthesis of green fluorescent proteins. 

Fredericamycin A (**15**) is a uniquely structured aromatic polyketide compound with excellent antitumor activity. Li et al. [41] identified the mutation of R444H in *rpoB* of the resistant *Streptomyces somaliensis* ZH66-RIF1 using a *rif* screen with 300 μg/mL of rifampicin, which resulted in activating a previously silent gene cluster. After fermentation optimization, an optimized yield of 679.5 ± 15.8 mg/L of **15** was obtained. MacMillan et al. isolated three new chlorinated alkaloids, inducamides A-C (**47**–**49**), from *Streptomyces* sp. SNC-109-M3, a *rif* mutant with a S442Y mutation in *rpoB*. Inducamides contain a 6-methylsalicylic acid unit and a tryptophan derivative, connected through an amide bond. Inducamide C (**49**) exhibited moderate cytotoxicity against NSCLC cell line HCC44 at 10 μM. 

Kuzuyama et al. screened a total of 164 *rif* mutants among 11 actinomycetes and identified one mutant TW-R50-13 with an *rpoB* H437Y mutation [87]. Isolation of two overproduced metabolites in TW-R50-13 and their structural elucidation led to the discovery of three methylbenzene containing linear polyketides **55**–**57**. Using the same approach, the authors screened a total of 114 *rif* mutants among 9 actinomycetes and identified one *Streptomyces* strain, S55-50-5, with the identical *rpoB* H437Y mutation [87]. This strain produced a novel isoindole-containing tetracyclic polyketide isoindolinomycin (**58**) with moderate cytotoxicity against the tested tumor cell lines and antibacterial activity against *Staphylococcus aureus*. 

In addition, ribosome engineering can also activate silent BGCs and discover new active compounds in other bacterial species or fungi. Ochi et al. [52] first introduced a rifamycin-induced *rpoB* mutation into the *B. subtilis* strain, which led to the production of a new aminosaccharide antibiotic 3,3’-neotrehalosadiamine (**31**), for which production is silent in the wild-type strain. In a metabolomic phenotype screening of *str* or *rif* mutants in a rare actinomycete *Nocardiopsis* sp. FU40 ΔApoS8, 311 unique metabolomic features were revealed by ultra-performance liquid chromatography ion-mobility mass spectrometry analysis [81]. This led to the isolation of five mutaxanthenes (**36**–**40**) with the unprecedented 12,12a-dihydro-1H-benzo(b)anthene–based scaffold in a *rif* mutant R4. Based on these data, Derewacz et al. suggested that activation of BGCs might be important cellular responses of certain antibiotic resistance mutations in the mutants [81]. Compounds extracted from fungi are an important source for new drug development [89,90]. By screening and isolation of neomycin-resistant mutants in marine-derived fungi, multiple active fungal natural products **41**–**46** and **50**–**54** were isolated from *Aspergillus versicolor* ZBY-3 or *Penicillium purpurogenum* G59, respectively [82,84]. 

## 4. Possible Mechanism of Action of Ribosome Engineering 

Based on the above analysis, ribosome engineering has found a broad utility to increase the yields of many microbial metabolites and to facilitate discovery of new natural products in *Streptomyces* and many other microbes. These studies not only generate high yielding industrial strains to produce affordable therapeutics but also identify dozens of natural products with unprecedented structures and promising biological activities. Biosynthetic studies of these natural products yield novel insights for catalysis and enzymology. In this section, we discuss the possible mechanisms involved in ribosome engineering. 

### 4.1. The Stringent Response, ppGpp, and Ribosome Engineering

In 1969, Cashel and Gallant first observed the presence of guanosine 5′-diphosphate 3′-diphosphate ppGpp, the signaling molecule of the stringent response, in starved *E. coli* [91]. The cellular centration of ppGpp is normally relatively low. Once stimulated by environmental stress such as nutrient starvation, its massive accumulation triggers an emergency response in cells. Since ppGpp was found to bind to RNA polymerases, Ochi et al. suggested that the RNA polymerases with certain mutations on *rpoB* may mimic the RNA polymerase bound with ppGpp in the wild-type *S. coelicolor* A3(2) [28]. The *relA* gene encodes the bifunctional (p)ppGpp synthase/hydrolase, while *relC* gene encodes the 50S ribosomal protein L11. In *relA-* or *relC*-knockout *S. coelicolor* A3(2) mutant strains, incapable of producing ppGpp and Act (<0.03 in OD633), they identified more than a dozen *rif* mutants with mutations in *rpoB* that produced higher levels of Act than the wild-type strains M400 and 1147, respectively (0.43 or 1.71 in OD633). Similarly, a *relC*-knockout mutant of *S. lividans* 1326 lost the ability to produce ppGpp and Act, while *rif* mutants with certain mutations in *rpoB* could restore Act production [92]. *Actinomadura* sp. ATCC 39727 (renamed as *Nonomuraea* sp. strain 39 727) contains duplicated *rpoB* alleles, in which one *rpoB*, rpoB^R^, harbored the H426N missense mutation [93]. The fact that the production of the glycopeptide antibiotic A40926 in *Nonomuraea.* sp. ATCC 39727 was not controlled by the stringent response and the presence of *rpoB* gene polymorphism in 5 out of 75 rare actinomycetes [94] is consistent with the proposal that certain *rif* mutants may mimic the stringent response. In *S. mauvecolor,* Ochi et al. further showed that the mutant RNA polymerases isolated from stationary-phase cells have enhanced affinity towards three *S. coelicolor* promoters ACTII-ORF4p, SIGNp1, and SIGNp2 in vitro [79]. Structure studies of the binding between these mutant RNA polymerases and promoters would provide further insights into these important interactions [95]. 

### 4.2. Ribosome Stability, Recycling, and Streptomycin-Resistance 

*Str* mutants may confer high or low levels of streptomycin resistance. High-level streptomycin resistance (Minimum inhibitory concentration (MIC) >100 μg/mL) may often result from K88E or K88R mutations in the ribosomal protein S12. In a salinomycin (**6**) producing industrial strain of *S. albus*, Ochi et al. observed that the mutant strain KO-600 (K88R) exhibited more than 3-fold enhanced protein synthesis activity during the stationary phase than the parental strain SAM-X, using an in vitro ribosome translation assay [45]. In addition, the K88R mutant ribosome appeared to be more stable than the wild-type ribosome in the presence of 0.8 mM Mg^2+^. In *S. coelicolor* A3(2), the K88E and P91S mutant ribosome also showed enhanced protein synthesis activity and could form a more stable 70S complex [96]. In addition, Ochi and coworkers simultaneously observed the overproduction of Act and increased expression of ribosome recycling factor in a strain of *S. coelicolor* with the K88E mutation in its S12 ribosomal protein [97]. They further demonstrated that overexpression of the *frr* gene encoding RRF in an *S. coelicolor* wild-type strain yielded higher protein synthesis rate and Act titer. Therefore, the increased translation in *str* mutants may significantly contribute to the observed titer increase. 

### 4.3. Sublethal Concentrations of Different Antibiotics and Ribosome Engineering 

Sublethal concentrations of different antibiotics, including lincomycin, streptomycin, erythromycin, have been used to induce the production of antibiotics. In a 1996 study, tetracycline, streptomycin, and hygromycin were able to induce Act production in *S. lividans* TK21 [15]. The maximum Act induction was observed when 10 or 15 μg/mL tetracycline or streptomycin was added to the cultures. At these concentrations, the growth of *S. lividans* was only slightly affected. In contrast, other tested antibiotics, including chloramphenicol, erythromycin, lincomycin, kanamycin, spectinomycin, fusidic acid, thiopeptin, thiostrepton, rifamycin, puromycin, ampicilin, and decoyinine, were unable to induce Act production. Interestingly, Ochi and coworkers also showed that lincomycin at subinhibitory concentrations (1/10 of the MIC of *S. coelicolor* A3(2)) increased Act overproduction by overexpression of the pathway-specific regulatory gene actII-ORF4 in *S. coelicolor* A3(2). Lincomycin (1/2 or 1/3 of the MIC of *S. lividans* 1326) could also lead to the production of novel congeners of calcium-dependent antibiotics in *S. lividans* 1326 [98]. At sublethal concentrations, these antibiotics might serve as the language to facilitate communication among the bacterial species [99]. It might be interesting to study the intrinsic relationship between antibiotic language and ribosome engineering. One hypothesis could be that the presence of these antibiotic stimuli in the natural environments might lead to the accumulation of spontaneous mutants, which were subsequently discovered using the ribosome engineering approach. 

## 5. Conclusions

To discover the next generation of medicines, the exploration of the biosynthetic potential in *Streptomyces* or other bacteria would require dedication, technology advancement, and intensive efforts. In the post-genomic era, many newly developed technologies have enabled the discovery of natural products from microbes or improvement of their production. Ribosome engineering remains a cost-effective way to discover new natural products and to enhance the titers of promising natural products, proteins, or biofuels in the engineered strains. The combination of ribosome engineering and other methods, such as genome shuffling, will be more effective to accelerate its development, especially for the development of high-yield industrial strains to produce natural products or other useful products. For example, ribosome engineering has only been used to improve the yield of a few industrial producing strains for the production of avermectins (**3**, **4**), salinomycin (**6**), vancomycin (**8**), and GE2270 (**18**). In addition, ribosome engineering could identify many useful mutant strains by the screening of a variety of antibiotics. The detailed mechanism of action of ribosome engineering in these mutants would need to be further elucidated. For example, structure studies of the interactions of the mutated RNA polymerases and their respective promoters would increase our understanding of the precise mechanisms of gene expression control. In the future, researchers may be able to activate certain BGCs or to improve the production of designed natural products in a more specific manner using ribosome engineering. 

## Figures and Tables

**Table 1:** Summary of *Streptomyces* strains used for ribosome engineering.

Antibiotic	Strain	Method	Mutation ^a^	(Fold)/(g/L) ^b^	Year/Ref.
Actinorhodin (**23**)	*S. coelicolor*	Str, Tet	- ^c^	-	1996 [15]
	*S. coelicolor*	Str	K88E	15 (2.8 OD600)	1997 [25]
	*S. coelicolor*	Par	P91S	5–21 (2.1 OD600)	2000 [26]
	*S. coelicolor*	Str, Gen, Rif	K88E	48 (6.88 OD633)	2001 [27]
	*S. coelicolor*	*relA* and *relC* mutants Rif	Q424L	>93 (2.79 OD633)	2002 [28]
	*S. coelicolor*	Str, Rif, Par, Gen	-	180 (1.63)	2008 [29]
	*S. coelicolor*	Str	R86P	55–106 (0.1338 ± 0.007)	2009 [30]
	*S. lividans*	Ery	-	6–8 (0.3)	2012 [31]
	*S. coelicolor*	Rif	S433L	42–55.5 (28.7 ± 1.3)	2013 [32]
Actinomycin D (**9**)	*S. antibioticus*	Str	-	5.2 (0.063)	1998 [33]
	*S.antibioticus*	Gen	-	4.1 (0.05)	2008 [34]
	*S. parvulus*	Str	K88R	2–10 (0.0328 ± 0.0086)	2009 [30]
	*S. antibioticus*	Str	K88R	7–10 (0.0471 ± 0.0044)	2009 [30]
	*S. parvulus*	Rif	D427V	1–2.2 (0.010 ± 0.001)	2013 [32]
	*S. antibioticus*	Rif	H437R	5–11 (0.086 ± 0.016)	2013 [32]
Avermectins (**3**,**4**)	*S. avermitilis*	*frr* overexpression	-	3–3.7 (>0.8)	2010 [35]
Avilamycin (5)	*S. viridochromogenes*	^60^Co γ-ray, GS, Str	K43N	36.8 (1.4)	2013 [36]
Chloramphenicol (**21**)	*S. coelicolor*	Str, Rif, HE	-	20–40 (−)	2011 [37]
Congocidine	*S. coelicolor*	Str, Rif, HE	-	20–40 (−)	2011 [37]
Daptomycin (**7**)	*S. roseosporus*	Ple	-	1.3 (>0.08)	2013 [38]
	*S. roseosporus*	Neo, Gen, Rif, Par, GS ^a^	-	4 (0.324)	2018 [39]
A21978C	*S. roseosporus*	Str, Reporter gene	K43N	2.2 (>0.12)	2012 [40]
Formycin A (**28**)	*S. lavendulae*	Str	-	5.2 (0.13)	1998 [33]
	*S. lavendulae*	Str	R440H	2.4–4.6 (0.055 ± 0.014)	2013 [32]
Fredericamycin A (**15**)	*S. chattanoogensis*	Str	-	26 (0.26)	1998 [33]
	*S. somaliensis*	Rif	R444H	3 (0.6795 ± 0.0158)	2015 [41]
Milbemycin (**19**,**20**)	*S. bingchenggensis*	CM, Str, UV	-	1.8 (1.45)	2009 [42]
Nosiheptide (**1**)	*S. actuosus*	^60^Coγ-irradiation, LiCl, Str, GS	K88R	9.2 (1.54)	2014 [43]
Oligomycin (**26**)	*S. avermitilis*	Str	K43M	20–40 (1.064)	2009 [30]
Rimocidin (**30**)	*S. rimosus*	Gen, Rif	-	2.5–6.2 (0.6731)	2019 [44]
Salinomycin (**6**)	*S. albus*	Str, Gen, Rif	-	2.3 (25)	2003 [45]
Sinefungin (**27**)	*S. incarnatus*	Rif, L-Arg	D427G	35 (>0.05)	2010 [46]
Streptomycin (**10**)	*S. griseus*	Gen	-	10 (0.3)	2008 [34]
	*S. griseus*	Rif	Q424K	2.4–6.0 (0.178 ± 0.027)	2013 [32]
Tiancimycin A (**17**)	*S.* sp. CB03234	Rif	L422P	40 (0.0225 ± 0.0031)	2016 [47]
					2018 [48]
	*S.* sp. CB03234	Str	K43N	45 (0.0137 ± 0.0003)	2019 [49]
Tiancimycins D (**14**)	*S.* sp. CB03234	Str	K43N	109 (0.0192 ± 0.0004)	2019 [49]
Toyocamycin (**16**)	*S. diastatochromogenes*	*frr* overexpression	-	1.46 (>0.6)	2014 [50]
	*S. diastatochromogenes*	Rif	H437Y	4.5 (0.68)	2016 [51]
Virginiamycin (**2**)	*S. virginiae*	UV, GS, Str	-	11.6 (0.251)	2018 [52]
6′-Deoxy-bleomycin Z (**12**)	*S. flavoviridis*	UV, Str, Gen, Rif	-	7 (0.07)	2018 [53]

The abbreviations are as follows: GS, genome shuffling; HE, heterologous gene expression; CM, chemical mutation; HEE, high energy electron; HT, high throughput. The abbreviations Ple, Neo, Ery, Gen, Kan, Lin, Par, Rif, Str, and Tet indicate resistance to pleuromutilin, neomycin, erythromycin, gentamicin, kanamycin, lincomycin, paromomycin, rifampicin, streptomycin, and tetracycline, respectively. ^a^ The strains with the highest titer of *rpoB* and *rpsL* mutation. ^b^ The term “fold“ was defined as the increased yield of the mutant strain compared to its parental strain, which was recalculated based on the cited articles. ^c^ The highest yield strain has no detected mutation within the *rpoB* and *rpsL* gene.

**Table 2:** Summary of non-*Streptomyces* mutant effective for antibiotic overproduction.

Antibiotic	Strain	Method	Mutation ^a^	(Fold)/(g/L) ^b^	Year/Ref.
Bacilysin (**25**)	*B. subtilis*	Str, Rif	K56R L467P	5–7 (0.0166 ± 0.0009)	2015 [54]
Erythromycin (**11**)	*S. erythraea*	Rif	S444F	4 (>1.5)	2009 [55]
	*S. erythraea* ^d^	Rif	H437R	4.0 (0.163 ± 0.034)	2013 [32]
FR900493 (**22**)	*B. cereus*	Str	- ^c^	7.2 (0.55)	1998 [33]
	*B. cereus*	Gen	-	2.7 (0.22)	2008 [34]
GE2270 A (**18**)	*P. rosea*	Gen, Str, Rif	-	1.8 (−)	2006 [56]
Norvancomycin	*A. orientalis*	Str, Rif, UV, HEE	-	1.4 (−)	2006 [57]
Pyrrolnitrin (**29**)	*P. pyrrocinia*	Str	-	10 (0.015)	1998 [33]
	*P.pyrrocinia*	Gen	-	5.3 (0.008)	2008 [34]
Vancomycin (**8**)	*A. orientalis*	Rif	S442Y	2.6–3.4 (0.27 ± 0.017)	2013 [32]
Violacein (**24**)	*E. coli*	Lin, Kan, HE	-	41 (−)	2011 [58]

^a^ The strains with the highest titer of *rpoB* and *rpsL* mutation. ^b^ The term “fold“ was defined as the increased yield of the mutant strain compared to its parental strain, which was recalculated based on the cited articles. ^c^ The highest yield strain has no detected mutation within the *rpoB* and *rpsL* gene. ^d^ *S. erythraea* indicates *Saccharopolyspora erythraea.*

**Table 3:** Yield improvement of primary metabolites, proteins, and fuels by ribosome engineering.

Miscellaneous Products	Strain	Method	Mutation ^a^	Fold/(g/L) ^b^	Year/Ref.
CITase	*P. agaridevorans*	Str, Rif	K56R, R485H	1100 (1104 ± 143 U/mL)	2018 [60]
α-Amylase	*B. subtilis*	Str	K56R	1.5 (4.0 U/mL)	2006 [61]
Xylanase	*S. viridochromogenes*	Str	K88R	1.14 (>60 U/mL)	2013 [62]
Vitamin B12	*P. shermanii*	Rif, Gen, Ery	H437Y, H447R	5.2 (304 ± 3 µg/L/OD600)	2017 [63]
ε-poly-L-Lysine	*S. albulus*	ARTP, Str, GS	- ^c^	1.71 (3.0) ^d^	2016 [64]
	*S. albulus*	HT, Par	-	1.45 (2.59)	2017 [65]
	*S. albulus*	Str, Gen, Rif	K108R	1.75–2.39 (3.83)	2017 [66]
	*S. albulus*	Str	E85G	1.79 (3.04)	2019 [67]
L-Isoleucine	*Corynebacterium glutamicum*	*frr* and *fusA* Overexpression	-	1.76 (28.5)	2015 [68]
Butanol	*Clostridium* *Saccharoperbutylacetonicum*	Str	K43N	1.6 (16.5)	2017 [69]
Ethanol	*K. variicola*	Str	K43N	1.3 (34)	2015 [70]

^a^ The strains with the highest titer of *rpoB* and *rpsL* mutation. ^b^ The term “fold” was defined as the increased yield of the mutant strain to its parental strain, which was recalculated based on the cited articles. ^c^ The highest yield strain has no detected mutation within the *rpoB* and *rpsL* gene. ^d^ This is the highest yield of the ε-poly-L-lysine in shake flasks. CITase: cycloisomaltooligosaccharide glucanotransferase.

**Table 4:** New natural products discovered through ribosome engineering.

Natural Products	Strain	Method	Mutation ^a^	Activity	Year/Ref.
Neotrehalosadiamine (**31**)	*B. subtilis*	Rif	S487L	Antibacterial	2004 [80]
Piperidamycins (**32**–**34**)	*S.* sp. 631689	Rif, Str, Gen	K88R	Antibacterial	2009 [79]
γ-Actinorhodin (**35**)	*S. coelicolor*	Rif	- ^b^	Antibacterial	2013 [32]
Mutaxanthenes (**36**–**40**)	*Nocardiaceae* FU40ΔApoS8	Rif, Str	-	-	2013 [81]
**41**–**46**	*Aspergillus versicolo* ZBY-3	Neo	-	Antitumor	2014 [82]
Inducamides A-C (**47**–**49**)	*S.* sp. *SNC-109*	Rif	X442F	-	2014 [83]
**50**–**54**	*Penicillium purpurogenum* G59	Neo, DMSO	-	Antibacterial Antitumor	2015 [84]
Fredericamycin A (**15**)	*S. somaliensis* SCSIO ZH66	Rif	R447H	Antitumor	2015 [41]
16 secondary metabolites	*S. coelicolor*	Rif, Str	-	Antibacterial	2015 [85]
**55**–**57**	*S.* sp. SANK 60404	Rif	H447D	-	2016 [86]
**58**, **59**	*S.* sp. SoC090715LN-16	Rif	H447Y	Antibacterial	2018 [87]
Cyclopentene derivatives	*S.* sp. HS-NF-1046R	Rif	-	-	2019 [88]

^a^ The strains with the highest titer of *rpoB* and *rpsL* mutation. ^b^ The highest yield strain has no detected mutation within the *rpoB* and *rpsL* genes.
